# “Every Cloud Has a Silver Lining”: How Three Rare Diseases Defend Themselves from COVID-19 and What We Have Learnt from It

**DOI:** 10.3390/clinpract14020048

**Published:** 2024-04-08

**Authors:** Martina Cacciapuoti, Ilaria Caputo, Lucia Federica Stefanelli, Paul A. Davis, Federico Nalesso, Lorenzo A. Calò

**Affiliations:** 1 Nephrology, Dialysis and Transplantation Unit, Department of Medicine, University of Padova, 35128 Padova, Italy; martina.cacciapuoti@aopd.veneto.it (M.C.); ilaria.caputo@unipd.it (I.C.); federico.nalesso@unipd.it (F.N.); 2 Department of Nutrition, University of California, Davis, CA 95616, USA; padavis@ucdavis.edu

**Keywords:** Gitelman’s syndrome, Bartter’s syndrome, Fabry disease, ACE2, Cathepsin-L, COVID-19

## Abstract

The process of SARS-CoV-2 infection, responsible for the COVID-19 pandemic, is carried out through different steps, with the interaction between ACE2 and Spike protein (S) being crucial. Besides of that, the acidic environment of endosomes seems to play a relevant role in the virus uptake into cells and its intracellular replication. Patients affected by two rare genetic tubulopathies, Gitelman’s and Bartter’s Syndromes, and a rare genetic metabolic disease, Fabry Disease, have shown intrinsic protection from SARS-CoV-2 infection and COVID-19 on account of specific intrinsic features that interfere with the virus uptake into cells and its intracellular replication, which will be reported and discussed in this paper, providing interesting insights for present and future research.

## 1. Introduction

SARS-CoV-2, an enveloped single-stranded RNA virus, belongs to the same lineage of SARS-CoV and MERS-CoV. Attachment of host cells with SARS-CoV-2 occurs through the interaction between viral spike proteins and the angiotensin-converting enzyme-2 (ACE2), the virus receptor, expressed on the host cell surface. The spike protein is a trimer with two domains, S1 and S2 [1]. The process of cell infection occurs in multiple steps: the N-terminal portion of S1 binds to ACE2, and the receptor transmembrane protease serine 2 (TMPRSS2) catalyzes the cleavage between S1 and S2, and finally the viral S2 protein undergoes a conformational rearrangement allowing the fusion between the viral and cellular membrane, and thus the release of viral RNA into the cytoplasm. In tissues where TMPRSS2 expression is low, SARS-CoV-2 enters the cell via endocytosis and host proteases such as cathepsin (Cat)-L that mediate the cleavage of spike proteins [2]. An essential requirement for this process is the acidic pH of endolysosomes [3]. The viral RNA translation in viral polyprotein takes place in the host cell ribosome where viral proteins are cleaved by the viral main protease (Mpro, also called 3-chymotrypsin-like protease (3CLpro) and papain-like protease (PLpro) to yield viral proteins effective for the viral replication transcription complex [2]. The N-terminal-glycosylation of ACE2 also plays a crucial role in the virus–cell interplay and virus uptake [4].

## 2. Mas Receptor and AT1R: The Good and the Bad

ACE2, a trans-membrane type I glycoprotein, catalyzes the cleavage of angiotensin I to angiotensin 1–9 and the conversion of angiotensin II (Ang II) in Ang 1–7 and is expressed in most human tissues including the heart, vessels, lung, and kidney. It is well known that Ang II can bind two different receptors, AT1R and AT2R, the former being responsible for the deleterious effects of the renin-angiotensin system (RAS) and the latter mediating opposite effects. In addition, Ang 1–7 binds the Mas receptor exerting anti-inflammatory, antithrombotic, antiproliferative, and vasodilatory effects, and the ACE2-Ang 1–7 pathway is considered the counter-regulatory arm of RAS [5]. 

After ACE2-SARS-CoV-2 spike protein interaction, ACE2 is internalized and its expression on the cell surface is downregulated, thus increasing serum levels of Ang II, determining a lower conversion of Ang II to Ang 1–7, and inducing hyperactivation of the Ang II signaling via AT1R. This so-called “Ang II storm” leads to an abnormal activation of the Ang II/AT1R cascade, thereby inducing organ damage via pro-inflammatory pathways [6].

Diabetes, hypertension, and a history of cardiovascular events leading to RAS dysregulation and ACE2 deficiency represent risk factors for increased disease severity in COVID-19 due to the imbalance between the Ang II-AT1R pathway and its ACE2-Ang1–7 counter-regulatory arm [6]. The role of ACE2 in SARS-CoV-2 infection arose doubts regarding the opportunity to discontinue treatments with ACE inhibitors or Angiotensin II receptor blockers, key in the treatment of hypertension, heart failure, and diabetes. It was assumed that ACEis and ARBs increasing ACE2 expression would have favored SARS-CoV-2 cell-entry. Indeed, SARS-CoV-2 causes ACE2 internalization thus lowering ACE2-mediated Ang II conversion into Ang 1–7 reducing its anti-inflammatory and antioxidant effects. In patients with RAS activation, RAS-blockers exert their beneficial effects by hampering Ang II/AT1R signaling, but also switching on the ACE2/Ang 1–7/MasR pathway [7]. This latter specific effect provides a reasonable explanation for the lack of worse outcomes in COVID-19 patients taking ACE inhibitors/Angiotensin II receptor blockers, which is confirmed by patients with Gitelman and Bartter syndromes—rare genetic tubulopathies, a human model of endogenous antagonism of Ang II signaling via AT1R—who have increased ACE2 and Ang 1–7 [7]. In addition, in vivo and in vitro experiments have shown that the ACE inhibitor captopril and the Angiotensin II receptor blocker candesartan increased the expression of ACE2 and Mas receptors in the lungs of young healthy rats, an effect that was even more evident in rats with metabolic syndrome, and also in human alveolar type-II pneumocyte cultures [7]. Moreover, RCTs provided strong evidence that RAS inhibitors can be safely used in patients with COVID-19, and that ACE inhibitors/Angiotensin II receptor blockers are not associated with poor COVID-19 outcomes [8].

## 3. Gitelman and Bartter Syndromes: Two “In Vivo” Models of SARS-CoV-2 Innate Protection

Gitelman and Bartter Syndromes (GS/BS) are two rare genetic tubulopathies characterized by metabolic alkalosis, hypokalemia (hypomagnesemia in GS), hyperactivation of RAS, and high Ang II and aldosterone level, yet normo/hypotension [9,10] and activation of the RAS counter-regulatory arm with increased ACE2 and Ang 1–7 levels, resulting in antiatherosclerotic, antiproliferative, and antifibrotic effects [5,11]. The Ang II-AT1R signaling pathway is indeed blunted in these patients, as they show a downregulation of the RhoA-Rho kinase (ROCK) pathway due to a higher expression of the Regulator of G-protein Signaling 2 that regulates the Ang II G protein coupled receptor signaling. The reduced activation of ROCK in GS/BS accounts for their increased vasodilation, antioxidant defenses, and antiatherogenesis, despite the increased levels of Ang II [5,11].

In the light of this evidence, and considering the critical role of ACE2-SARS-CoV-2 interplay in COVID-19, we thought that Gitelman and Bartter patients, with their increased ACE2 level, could be a good model to investigate susceptibility to developing COVID-19 infection. Three surveys in our cohort of Gitelman and Bartter patients, in the hot spots for COVID-19 in Northern Italy, assessed their rate of SARS-CoV-2 infection. The first, in early 2020, showed none of the 128 patients of our cohort with COVID-19, significantly lower than the prevalence of COVID-19 in the general population [12]. The second, in Spring 2021, showed eight patients out of one hundred and twenty-eight with COVID-19, four asymptomatic, and four with very mild symptoms [13]. White the third survey, December 2021–January 2022, during the spread of the more-infectant SARS-CoV-2 Omicron variant, we reported fourteen patients with COVID-19, nine asymptomatic and five with very mild symptoms, four of them with no SARS-CoV-2 vaccination [13]. The vaccination campaign may have contributed to reducing the rate of COVID-19 in these patients. However, it appeared that in patients with Gitelman and Bartter syndromes, SARS-CoV-2 infection was asymptomatic or pauci-symptomatic, which led us to investigate the mechanistic basis of this surprising result.

Gitelman and Bartter patients have higher expression of ACE2 and Ang 1–7, which is also an effect of ACE inhibitors/Angiotensin II receptor blockers, thus supporting the RASi protective effect in SARS-CoV-2 infection in patients with hypertension or diabetes [7,11]. In addition, as the ACE2 glycosylation and Cat-L activity require an acidic environment in intracellular organelles (endosomes, Golgi, and transGolgi network), the likely altered (less acidic) intracellular pH, due to the Gitelman and Bartter patients’ metabolic alkalosis, is not favorable for these processes to be carried out [13]. This mimics the effect of chloroquine/hydroxychloroquine in SARS-CoV infection, as it interferes with terminal glycosylation of ACE2 while leaving ACE2 membrane expression unaltered. When chloroquine was added after infection, it rapidly rose the intracellular pH and undermined fusion events between the virus, transGolgi network, and endosomes, inhibiting the virus entry and its intracellular replication. This was also proven in SARS-CoV-2 cells [14], although no evidence of clinical benefit in treating COVID-19 hospitalized patients with hydroxychloroquine was provided. 

The above-mentioned intracellular organelles pH mechanistic hypothesis is supported by the results of our study in a cohort of Gitelman and Bartter patients performed to assess the levels of ACE2 and its glycosylation state and Cat-L activity. We found that Gitelman and Bartter patients, compared to healthy subjects, had significantly higher non-glycosylated ACE2 levels and lower Cat-L activity, which inversely correlated with their blood bicarbonate [13]. In addition, as RhoA/ROCK pathway activates Nf-κB, reduces eNOs activity, and increases ROS production [11], the upregulation of ROCK signaling might contribute to the induction of acute respiratory distress syndrome (ARDS) in COVID-19. ROCK inhibitors have in fact been shown to be effective in the treatment of ARDS [15], and none of our GS/BS patients, who have blunted RhoA/ROCK pathway [11], were hospitalized for SARS-CoV-2 pneumonia [14]. It is therefore reasonable to assume that in Gitelman and Bartter patients, both their endogenously higher ACE2 non-glycosylated isoform, the reduced Cat-L activity, and the blunted ROCK signaling might give a mechanistic explanation to the reduced susceptibility of these patients to COVID-19 [13,16].

## 4. Fabry Disease and SARS-CoV-2

After SARS-CoV-2 entry into the cells, it is discharged into the cytosol from endolysosomes or is dispatched for degradation in lysosomes. Moreover, some coronaviruses, including SARS-CoV-2, are enabled to escape endolysosomes and replicate in autophagosome-like structures in the cytosol [3].

Fabry disease (FD) is a X-linked inherited disorder characterized by the deficiency of alfa-galactosidase A (α-GalA) resulting in the accumulation of globotriaosylceramide (Gb3) and globotriaosylsphingosine (lysoGb3) in lysosomes [17]. This results in impaired autophagy, leading to mitochondria and endoplasmic reticulum dysfunction [18]. Oxidative stress also contributes to the damage [18]. Clinical manifestations of FD include kidney disease with proteinuria, hypertrophic cardiomyopathy, arrhythmias, coronary artery stenosis, and cerebrovascular disease, male patients presenting with the most severe clinical symptoms [17].

Recently, a very low rate of COVID-19 was reported in FD patients. Only 1 patient out of 234 was, in fact, reported with a very mild form, and 2 were asymptomatic [19]. It was suggested that the molecular basis of this “protection” from COVID-19 was to be sought in the impaired lyososomal functioning due to glycosphingolypids accumulation into the cells, leading to an unfavorable host cellular environment that hampers virus infection and replication [19]. This was confirmed in our cohort of FD patients: only 1 out of 40 enzymatic-replacement-therapy-treated FD patients tested positive for COVID-19 with minor symptoms. In addition, we showed in these patients higher non-glycosylated ACE2 levels, lower Cat-L activity, and a lower glycosilated/non glycosylated ACE2 ratio compared to healthy subjects [20], giving a molecular rationale for this “protection”. 

**Figure 1:**
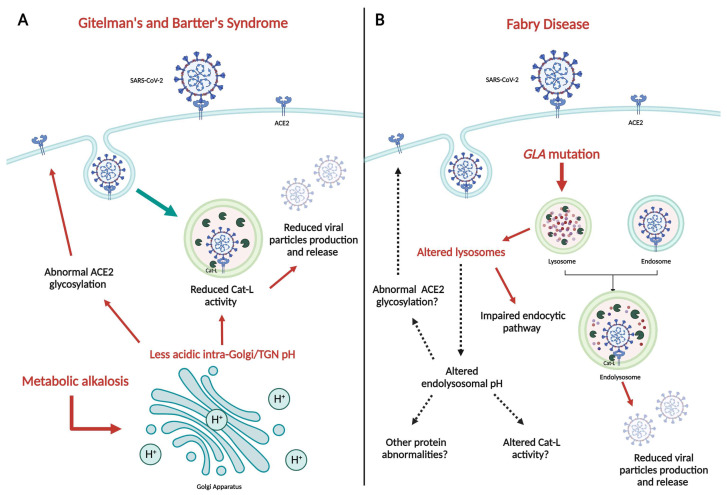
Cellular mechanisms for protection from SARS-CoV-2 infection in GS/BS and FD patients. Metabolic alkalosis induced pH alteration in the Golgi and transGolgi network; trafficking lowers endosomal Cat-L activity and impairs ACE2 glycosylation process in GS/BS patients (**panel A**). Mutation in the GLA gene leads to abnormal lysosomal storage disorder which induces impaired endocytic pathway. Altered lysosomes might modify endolysosomal pH and, as a consequence, impair glycosylation mechanisms, Cat-L activity, and induce protein abnormalities (dashed arrows). All these mechanisms lead to reduced SARS-CoV-2 viral particles production and release in Fabry disease (**panel B**). Created with BioRender.com (accessed on 6 December 2023).

In conclusion, GS/BS and FD have shown a natural protection from COVID-19, likely due to the increased ACE2/MasR signaling activity, the metabolic alkalosis, and the lysosomal dysfunction (Figure 1). 

This is also the mechanistic rationale of the new antiviral Paxlovid action, which inhibits proteins involved in lysosomal processes [21]. Finally, GS/BS via their blunted Ang II/AT1R signaling and ACE2/MasR activation, have also contributed to provide evidence against the discontinuation of the use of ACE inhibitors and Angiotensin II receptor blockers in SARS-CoV-2 infected patients [7].

## Data Availability

Not applicable.
